# Dynamic complexity of moral motivation: the roles of duty and self-interest

**DOI:** 10.3389/fpsyg.2026.1759470

**Published:** 2026-09-03

**Authors:** Ulas Kaplan

**Affiliations:** State University of New York at Oswego, Oswego, NY, United States

**Keywords:** care, duty, self-interest, dynamic systems, justice, moral motivation

## Abstract

Insights from moral philosophy are identified and presented to explicate the conception of moral motivation as a complex dynamic system based on multiple motivational tendencies that have competitive and cooperative relationships with each other. As part of process-relational metatheory, the perspective of complex dynamic systems is applied. Self-interest and duty are conceptualized as motivational tendencies. Two specific forms of duty are examined: justice as duty and care as duty. The psychological construction and regulation of duty and self-interest are discussed as fundamental processes in the dynamic system of moral motivation. This view has important theoretical and practical implications in terms of understanding and addressing real-life moral complexity.

## Introduction

Morality involves issues of justice, rights, duties and obligations (Dewey and Tufts, 1910; Kant, 1996; Kohlberg and Candee, 1984). Moral judgment is a judgment about justice, as it often includes an evaluation of action regarding rights, duties, obligations and responsibilities that people have as social beings (Colby et al., 1983; Kohlberg, 1984). In this sense, moral judgments are mainly about *exercise of power*, with direct or an indirect impact on one or more individuals. Hence, moral judgment is about the appropriateness, acceptability or the necessity of a particular exercise of power in a specific social context (Hauser, 2006). It is a premise of the present paper that moral judgments and action have their psychological bases in multiple motivations. Each motivation is a constellation of emotion-cognition relationships. Multiple motivations compete and cooperate with each other in the dynamic system of moral motivation toward the emergence of judgments and actions. (Kaplan, 2014, 2017; Kaplan and Fang, 2025)

Moral judgment is a component of moral cognition, and motivation is a function of the human will. Because thinking and willing are interconnected (Hegel, 1952), all cognition is motivated. “Man cannot use his theoretic faculty or think without will, for in thinking we are active” (Hegel, 1952, p. 22). It follows that moral cognition, including moral judgment, is motivated. By contrast, in the psychological study of morality, motivation has been traditionally reserved for the generation of overt moral behavior (Blasi, 1990; Nucci, 2002; Nunner-Winkler, 1998). However, judgment also emerges from a process of motivation. “The act of judging is not merely an active experience at large, but one which requires specific motivation” (Dewey, 1903, p. 125). Therefore, studies of moral judgment can benefit more directly from motivational and philosophical approaches and insights. While there is a recognition that moral motivation is central for both moral thinking and action (Carlo and Edwards, 2005), such a comprehensive view is more implicit than explicit in moral psychology.

Motivation is about a preference toward a particular experience (e.g., perception, action, cognition) relative to others. Such a preference reflects the motivational value of experience. The origin and operation of any motivation indicates that the organism values a particular experience, and prefers it more strongly than other possibilities (Thelen and Smith, 1994). Hence, moral motivation involves particular issues, sentiments, needs and goals that are experienced to be relatively valuable by the individual while making evaluations about rights, duties and obligations in the process of moral adaptation to the intrapsychic and social environment. There is no logical or phenomenological necessity for this preferential experience to be limited to the emergence of overt behavior. It also applies to the generation of forms of cognition including the emergence of moral judgment. “There must be some stimulus which moves to performing this particular sort of act rather than some other. Why engage in that particular kind of activity that we call judging?” (Dewey, 1903, p. 125).

The present perspective is consistent with the process-relational metatheoretical perspective (Overton, 2013, 2015) that aims to transcend the dichotomies of the split metatheory. Accordingly, judgment and action are closely intertwined, although they can be differentially examined. Motivation is a process of generating not just overt action, but also judgment, as moral cognition is motivated (Kaplan and Tivnan, 2014a,b,c). Likewise, the goal-directed view of cognition broadens the notion of action, bringing it closer to cognitive activity. Accordingly, “a concept must be motivated… and a motive cannot help bring forth the concept that can satisfy it” (Lewis, 2010, p. 184). Moral cognition, including the use of concepts, evolves in both microdevelopment and macrodevelopment iteratively in the pursuit of goals, as multiple emotions and moral motivations operate over time.

## Iterative and contextual nature of moral motivation

According to the temporal, iterative and contextual nature of moral motivation, an individual’s present moral motivational state (with its interconnected cognitions and emotions) influences the next motivational state, including changes in cognitions and emotions. These changes occur rapidly in the context of an actual or imagined social interaction, through appraisals that mostly operate automatically and intuitively. This iterative account of moral judgment, action and motivation is consistent with Dewey’s (1903) emphasis on the recursive nature of moral judgment:

Judgments are “moral” [vs. merely intellectual] in logical type so far as the presence of activity in affecting the content of judgment is seen consciously to affect itself an object of judgment whose determination is a prerequisite for further successful judgments. (p. 139).

Thus, the emerging moral judgment is not isolated in time and space, merely as a function of stable attitudes or present environmental conditions (e.g., the type of moral dilemma). Rather, it builds on and emerges out of the preceding motivational states of the organism, as well as serving toward the emergence of new judgments, “for the sake of the development of further experience. This further development is change, transformation of existing experience, and thus is *active*” (Dewey, 1903, p. 139).

The motivational state at a given time point can be represented by multiple appraisals and multiple emotions. In this motivational process, the elements of social context are dynamically related to intrapsychic factors including moral emotions and cognitions (Kaplan, 2017). It follows that “the social and cultural expectations become part and parcel of the felt emotions and of the activities that communicate those felt emotions to others” (Thelen and Smith, 1994, p. 320). This view resonates with a recent approach to character development according to RDS: “the conceptual emphasis in RDS theories is placed on mutually influential relations between individuals and contexts, represented as individual← → context relations” (Lerner and Schmid Callina, 2014, p. 325).

This reciprocal determination between the individual and context was emphasized by Dewey and Tufts (1910) as the “twofold aspect of life and conduct” (p. 2). Accordingly, moral inquiry is the study of “the inner process as *determined by the outer conditions or as changing these outer conditions*, and the outward behavior or institution *as determined by the inner purpose, or as affecting the inner life*” (p. 3).

## Instrumental-communicative and expressive-constitutive aspects of moral motivation

From a process-relational metatheoretical perspective, Overton (2006, 2015) distinguished two functions of human behavior. The instrumental-communicative dimension is about the adaptive purposes that behavior serves. The expressive-constitutive dimension is about the underlying system of organization that behavior reflects. Moral motivation as a process of moral adaptation represents the instrumental-communicative function of behavior. According to this function, moral motivation serves human adaptation by generating moral understanding, judgment and action. In this sense, moral motivation is goal oriented. Moral motivation as a dynamic system represents the expressive-constitutive function of behavior. According to the expressive-constitutive function, moral judgment and action reflect an underlying system of multiple moral motivations. In this sense, moral motivation represents the state of the organism at a given point in time.

## Self-organization and self-regulation in moral motivation

Moral motivation has been defined as the dynamic developmental process of self-organization and self-regulation of cognitive and emotional elements out of which moral judgment and action emerge (Kaplan, 2017). This conception is based on the perspective of Complex Dynamic Systems (CDS), as part of the process-relational metatheory. Accordingly, multiple motivations are connected to each other through supportive and competitive relationships in the dynamic system of moral motivation. This view reflects the dynamic nature of moral motivation as well as its central role in the emergence of moral judgment and action. A major function of moral motivation is to solve adaptive problems through relatively adequate emotional, cognitive and behavioral responses to the demands and realities of the social world (e.g., other people’s feelings), and the intrapsychic world (e.g., one’s own conscience). The present article builds on this view in order to specify and shed light on the construction and regulation of self-interest and the sense of duty in the motivation of moral judgment and action by incorporating insights from (a) moral philosophy, (b) moral psychology, and (c) the process-relational metatheory.

Moral motivation is both self-organizational and self-regulational. These two processes are dynamically related in the motivation of moral judgment and action. Self-organization represents spontaneous activity of a system through emergent relationships between its components. Based on self-organization, new experiences (such as a new type of moral understanding) emerge through relationships between simpler components of the system. Self-regulation represents “human agency in the context of individual desires, purposes, needs, goals and identity, other people, the physical ecology, and culture” (Lerner and Schmid Callina, 2014, p. 329). In the dynamic motivational process, self-regulatory influences (representing agency and intentional change) become integral parts of the self-organization of moral emotions and cognitions.

It is important to clarify that in the self-organization and self-regulation of moral motivation, the specific ways in which the individual shapes moral experiences may not always represent development or the greater good. This point is about the distinction between *is* and *ought* (Kohlberg, 1971). That is, while there is an essentially developmental and adaptive quality to moral self-organization and self-regulation, not all attempts enhance development or adaptation. Moral motivation is the process of self-organization and self-regulation, out of which judgments and actions emerge (Kaplan, 2017). In this conception, moral motivation is not limited to the generation of prosocial behaviors. It is the overarching process for the emergence of a wide range of moral actions and judgments. Thus, as cognition is motivated, moral decision making is a motivational process.

With the foregoing broad construal of moral motivation, it becomes clear that the ongoing outcomes of motivation can be highly variable in their relative qualities of benevolence or vice. In the constructive-developmental process of responding to mental demands of modern life (Kegan, 1994), individuals can display a wide range of behaviors, including destructive ones (Zimbardo, 2004). Because moral activity is both psychologically and contextually constructed, and thus highly variable; there can be relatively destructive or maladaptive actions and judgments arising from this process of adaptation. This insight is consistent with the developmental view and evidence of defense mechanisms (Vaillant, 1993), which can function in maladaptive ways despite their originally adaptive orientations.

The present conception of moral motivation builds on the emphasis of the *Relational Developmental Systems* (RDS) perspective (Overton, 2013) on both self-organization and self-regulation as fundamental qualities in human development. According to RDS, the human organism is “a spontaneously active, self-creating (autopoetic, enactive), self-organizing, and self-regulating nonlinear complex adaptive system” (Overton and Lerner, 2014, s. 64). Self-organization is a bottom-up process by which system components form spontaneous relationships. Self-regulation is an intentional process by which the person actively manages her own experience.

The importance of self-regulation in morality was emphasized by Aristotle (1999) in his account of the temperate human being who is able to regulate desires in accordance with reason, in harmony with the “rational principle” (p. 53). This is a principle that enables individuals to detect and avoid excess and defect, and to choose moral virtue in action, namely the intermediate between vices. Right action and judgment are those based on the facts of the situation according to this principle. This is a tendency to judge and act “according to the merits of the case and in whatever way the rule directs” (p. 45). This tendency is contrasted by actions and judgments that lean heavily toward excess or defect as a result of bias or impulse.

Moral self-regulation can be viewed as a direct exercise of will. It involves the possibility of top-down influence of higher-order experiences such as sense of purpose, intentional goals, awareness, habits, identity, character and moral principles on real-time moral thinking, feeling, judgment and action. Needs, goals and decisions can have strong self-regulatory influences on emotions, cognitions (including selective attention and perception), intuitions and behavior within the moral motivation process. Of particular importance to moral functioning is the strong possibility that self-regulation involves awareness of oneself as a social being. The role of social cognition makes self-regulation critical for moral judgment and motivation.

The present emphasis on self-regulation as one of the core features of moral motivation is consistent with the view of Narvaez on moral character development in terms of moral expertise. According to Narvaez (2008), moral experts “cultivate self-regulation that leads them to prioritize and deepen commitment to ethical goals” (p. 312). Consistently, self-regulation must be supported as one of the steps of Integrative Ethical Education (IEE), which Narvaez proposed as a model to facilitate character development. According to IEE, supporting self-regulation involves facilitating the coordination and integration of intuitive and deliberate moral functioning, such as conscious recognition and constructive use of moral intuitions. This emphasis reflects the instrumental-communicative (Overton, 2006) function of moral motivation.

From the present CDS view, character is not a fixed state, but a dynamic characteristic that represents both change and stability. As moral identity, character has an important influence on moral judgment (Kaplan, 2017). This account is consistent with Dewey’s (1903) perspective, according to which character refers to a “complex continuum of interactions in its office of influencing final judgment” (p. 126). In Dewey’s view, these interactions represent relations of habits to attitudes and other habits. Such dynamics of individual’s character are not “necessarily moral in themselves, are yet indispensable requisites for full morality” (Dewey and Tufts, 1910, p. 39). By full morality Dewey and Tufts (1910) meant a level of experience according to which “a man not only intends his acts definitely, he also values them as what he can do ‘with all his heart’” (p. 39). Consistently, according to Kant (1996), the ideal state of moral motivation is “the subjection of the heart to duty” (p. 264). Discussing the possible use of an example of this phenomenon to facilitate moral education and development, Kant stressed that “virtue is here worth so much, only because it costs so much, not because it brings any profit” (p. 265). Kant’s emphasis on the transformative value of duty resonates with Hegel’s (1894) view of duty in terms of the person’s identity: the “underlying essence” of duty is experienced as the individual’s “own very being” (p. 277).

## Multiplicity and complexity of moral motivation

In contrast to Kant’s aforementioned characterization of pure virtue, “our motivations rarely are pure gold. They are usually alloyed with one of several metals less noble than what is visible” (Horney, 1950, p. 125). This mixture was also emphasized by Nietzsche (1986, 2002) in his dialectic approach to morality beyond the dichotomy between good and evil. Dewey and Tufts (1910) identified a similar dialectic in the complexity of collective moral motivation in their account of processes as both rationalizing and socializing agencies: work (industry), art, and war. “All these, like craft, may be used for unmoral or even immoral ends, but they are also highly important as factors in an effective moral personality,” requiring and facilitating in-group cooperation (Dewey and Tufts, 1910, p. 42). Likewise, according to Greene (2013), the human brain that has evolved to solve the problem of cooperation (Me vs. Us) by subordinating individual desires to group norms, is the same brain that creates and perpetuates the conflicts between groups (Us vs. Them). This can be seen as a supporting example for the human brain’s possibility and capacity for *parallel processing*; namely the simultaneous operation of multiple, competing tendencies. Consistently, according to the present CDS view, multiple moral motivations compete and cooperate (Kaplan, 2017; van Geert, 1993) in their striving for more power and fuller expression in the life of the individual. Moral development involves increasingly *adequate* (Kohlberg and Mayer, 1972) regulation of multiple motivations.

From a CDS perspective, Kaplan and colleagues reformulated Kohlberg’s stages of reasoning as dynamic structures of motivation that operate together in multiplicity. They found evidence that multiple stages structures tend to function in the dynamic system of moral motivation, in ways that are contextualized, that is, contextually variable. Different contexts of moral decision making (different moral problems) were associated with different combinations of multiple stages in terms of their relative strengths (Kaplan and Tivnan, 2014a, 2014b; Kaplan et al., 2014). Furthermore, even a single moral judgment represented both variability and developmental order. Thus, from a CDS perspective, people are not at a stage; rather, multiple stage structures operate within person as motivational tendencies. The present study extends this viewpoint beyond the reformulation of Kohlberg’s stages into broader categories of self-interest and duty, which are constructed and variable individually and contextually.

Moral motivations are energetic tendencies that operate within the psyche composed of simpler emotions and cognitions that are interconnected. Each moral motivation demands recognition and realization through manifestation in moral judgment and action. This explanation is consistent with the contention of Dewey and Tufts (1910) that “interests in wealth, in knowledge, in power, in friendship, in social welfare, make demand for recognition in fixing upon what is good” (p. 3). In this sense, each motivation is governed by a life principle of *will to power* (Nietzsche, 1968), that is toward mastery and increase in power. For example, what Nietzsche (1968) identified as the *herd morality*, characterized by blind conformity to authority and to community, is itself governed by will to power: “*the herd instinct*… wants to be master: hence its “thou shalt!”—it will allow value to the individual only from the point of view of the whole” (p. 157). This dynamic also applies to qualities that are constructed and admired as *virtue* by a given society or an individual at a given period in time and history. According to Nietzsche, “virtues” are also governed by *will to power* in their nonvirtuous attempts to survive, prevail and dominate. This tendency can be viewed as a characteristic of each moral motivation operating within the psyche.

Kant (1996) expressed and agreed with a common sentiment of philosophers that real-life human conduct does not represent the purity of moral motivation based on duty: “though much may be done *in conformity with* what *duty* commands, still it is always doubtful whether it is really done *from duty* and therefore has moral worth” (p. 61). Kant’s argument rests in his view of the constitution of a human being. That is, human existence in time and space always involves some dependence on the laws of nature based on sensory conditions. Nietzsche (1986) developed a similar argument: “A being capable of nothing but unegoistic actions is more fabulous than the phoenix… How could the ego act without the ego?” (p. 71).

The foregoing ideas of Kant and Nietzsche were corroborated by Kohlberg’s (1971) observations when he referred to Kantian and utilitarian principles:

We have never encountered a live human being who made moral judgments in terms of principle in this sense… we do find people judging in terms of principles in a weaker sense… and these principles may not be definitive of a choice in all situations. (p. 219).

Similarly, Kant (1996) expressed that “being a creature” implies being “always dependent” on conditions of satisfaction. Hence, the human being “can never be altogether free from desires and inclinations which, because they rest on physical causes, do not of themselves accord with the moral law” (p. 207).

If Kant’s admission—about the improbability of human action being carried out solely from duty, without the mixture of personal incentives—is valid, then there are two possibilities. Either a sense of duty is almost never a motivating force for human action, or it operates in conjunction with other, less inclusive, more self-centered motives. If the former option was the case, duty would be a meaningless and empty notion that has no relevance to human affairs. By contrast, duty is a universal experience that human beings strive to fulfill in their daily living, including duties toward others (e.g., care as duty) and duties toward oneself (e.g., self-discipline in cultivating and developing one’s own talents and skills). Motivation for such duties sometimes conflict, sometimes cooperate with other motives such as self-interest. Therefore, the former possibility is not plausible, and the latter possibility is likely the case. That is, people are likely to act both with a sense of duty and concern for self-interest in various degrees. A sense of duty may frequently be a motivating force, but often not the only determining cause of the human will in the process of carrying out specific actions. It follows that daily human actions involve multiple motivations: self-interest and duty are likely to co-exist and even cooperate in the emergence of a single action. Self-interest may be reconstructed and constrained by duty; and the sense of duty may be revised as a result of concerns for self-interest.

From the present CDS perspective, the presence and prevalence of self-centered motives do not eliminate motivations based on duty; rather, their relationships with each other constitute and shape moral motivation. Moral psychological experience involves multiple motivational forces, operating simultaneously. There is a dialectic and dynamic motivational process by which these forces operate and relate to each other in the making of judgment and action. This notion was asserted by Nietzsche (2002) in *Beyond Good and Evil*: “We modern men are determined by a *diversity* of morals; our actions shine with different colors in turn, they are rarely unambiguous,—and it happens often enough that we perform *multi-colored* actions” (p. 110).

## Connections and distinctions between moral psychology and philosophy

As a philosopher, Kant’s primary concern was not about explaining how moral action occurs. Rather, he was mainly interested in explaining how human beings ought to act, namely discovering and articulating the highest principle of morality. Therefore, he insisted on the moral purity of actions, that is, their emergence merely out of respect for the moral law as the standard by which they can have moral worth. He stressed that moral philosophy, the account of what *ought* to happen, should not be based on experience, which is often subject to motives of self-interested motives. Rather, he argued that moral philosophy should be based on reason, which “independently of all appearances commands what ought to happen.” According to Kant, the command of reason is duty in its purity (as in friendship with utmost sincerity), which is valuable and important as what is morally right, even if this model of highest morality may not have been observed in experience. As the essence of morality, duty “lies, prior to all experience, in the idea of a reason determining the will by means of *a priori* grounds” (Kant, 1996, p. 62).

According to Kant, the moral law not only provides a standard for evaluating one’s conduct (and hence recognizing our imperfection, guarding against self-conceit), but also an ideal to strive for however impossible it may be to reach in our lifetime. Hence, the moral law provides a developmental vision to guide and measure our progress. Consistently, according to According to Kohlberg (1971), “ethical principles” constitute the “the end point” of moral development (p. 155).

This aim and priority of Kant should be taken into account in any evaluation of his philosophy. Critiques of and judgments about Kant’s philosophy from a merely psychological viewpoint are likely to be narrow, decontextualized and misleading. Such a viewpoint would make judgments about Kant’s work as if he was primarily concerned with explaining how people act. This is a major aim of moral psychology. This difference in priorities notwithstanding, a sound moral psychology can and must be grounded in moral philosophy. As Kohlberg (1971) asserted “the epistemological blinders psychologists have worn have hidden from them the fact that the concept of morality is itself a philosophical (ethical) rather than a behavioral concept” (p. 152): close connections between moral psychology and philosophy are likely to benefit both disciplines.

## The construction and regulation of self-interest in moral motivation

In human evolution, morality has a foundation in “the necessary activities of existence,” which “start the process” of moral development (Dewey and Tufts, 1910, p. 39). This evolutionary basis in terms of “the struggle for existence” (p. 39) is not lost to the contemporary human being; it is still operative in the contexts of modern life. Consistently, the motivation of self-interest represents the desire to maximize personal capabilities and welfare, and minimize personal costs, including the motivation to avoid pain and increase pleasure (Aristotle, 1999).

As a motivational tendency, self-interest prioritizes the individual’s personal welfare (Kant, 1996). Rousseau (1978) characterized this motivation as the personal will, preoccupied with one’s own advantage; distinguishing it from “the will of the people or the sovereign will,” which represents the common good in society (p. 43). According to Schopenhauer (1903), this is the motivation of *egoism* (pp. 157–158). Hegel (1952) construed it broadly, including “needs, inclinations, passions, opinions, fancies,” the satisfaction of which is pursued and experienced as “welfare or happiness” (p. 118). For Bentham (1823), self-interest is a ubiquitous motive to pursue pleasure and avoid pain. According to Mill (2009), this motive includes the universal human need for safety.

Both in theory and personal experience, self-interest can be conceived and constructed at various levels of comprehensiveness. Broadly, it can be seen and experienced as “acting so as to maximize positive emotions (e.g., pride) or minimize negative emotions (e.g., guilt),” whereas a narrow conception “requires that the interests motivating people be material ones (e.g., economic profit)” (Miller, 1999, pp. 1053–1054). According to the present perspective, the construction of each motivational tendency, including self-interest, varies substantially within person (across time and contexts) and among individuals.

Strong operations of self-interest were identified and discussed by Aristotle (1999) as self-indulgence and incontinence. Compared to incontinence, self-indulgence represents a more extreme type of self-interest. The self-indulgent human being is characterized as one who “pursues the excesses of things pleasant, or pursues to excess necessary objects, and does so by choice, for their own sake and not at all for the sake of any result distinct from them” (p. 116). Thus, self-indulgence is characterized by a deliberate pursuit of excessive self-interest, as this is all he knows. This is the opposite of temperance. On the other hand, the incontinent person is someone who lacks self-regulation, and acts irresponsibly and impulsively as he is easily driven by passions, even though he is capable of acting otherwise or he knows better. The self-indulgent person has a relentless conviction to pursue passions and pleasures. Hence, Aristotle associated this state with vice. The incontinent person on the other hand has more constructive intentions as well as higher self-awareness, but is not strong enough to resist temptation.

In Plato’s (1908) Republic, this motivation of self-interest was contrasted by “virtue and wisdom” (p. 202), and described in the possible experiences of politicians who “go to the administration of public affairs, poor and hungering after their own private advantage” (pp. 202–203).

The motivational process of forming a moral judgment and carrying out an action, as well as the emerging judgment and action are influenced by individuals’ existing goals as well as sociomoral attitudes (e.g., toward political issues and actors in public life), which are subject to changes over time. As Dewey (1903) pointed out, “every judgment… involves the functioning of an interest or end and the deploying of habits and impulsive tendencies (which ultimately involve motor adjustments) in the service of that interest” (p. 124). Significant and relevant goals are likely to have constraining influences on moral motivation by increasing the salience and significance of contextual factors that may facilitate or undermine goal pursuit and attainment. The kinds of judgments and actions that emerge in this process will be sensitive to these goals. Goals represent the interest of the organism. Ongoing needs, goals, desires and convictions constitute self-interest (Greene, 2013; Haidt, 2012), which forms the background in the context of which moral judgments emerge. This background can be considered as the intrapsychic context in which the judgment takes place. This context includes “the habits and impulsive tendencies of the one who judges” (Dewey, 1903, p. 126). Changes in this intrapsychic context can alter the judgment that emerges. “Change the interest or end, and the selected material (the subject of the judgment) changes, and the point of view from which it is regarded… changes also” (Dewey, 1903, p. 124). In turn, the emerging judgments and actions may have an influence on goal pursuits, including the possibility of revising existing goals. Consistently, Bocian et al. (2020) emphasized both personal benefits and group interests as factors by which egocentrism influences moral judgment.

## The unity of moral judgment and action in a developmental process

In the cognitive-developmental study of morality, moral judgment has been identified as *deontic choice* (Kohlberg and Candee, 1984). This concept represents individuals’ judgment of what the right thing is in a moral dilemma. In describing a result of a study by McNamee (1977), Candee and Kohlberg (1987) pointed out that “although all subjects tended to agree that it was right to disrupt an experiment to help a distressed victim, a greater proportion of subjects at each higher moral stage actually did so” (p. 556). In this experiment, 57% of the participants did not help the victim, and 72% reported in an interview after the encounter that it was the right thing for them to help (McNamee, 1977). Using the perspective of the old viewpoint, the interpretation of this finding would be that there was strong inconsistency between moral judgment (deontic choice) and action. Furthermore, this inconsistency was variable in a way that could be explained by moral stage. At higher stages stronger consistency was found between moral judgment and action.

These findings can be reinterpreted from the present CDS perspective, which proposes a unity of judgment and action, in a way that reflects real-life moral experience more closely. When the majority of participants in McNamee’s experiments “acted” not to help a victim in need, when they encountered him during the experiment, they “judged” that it was the right thing not to help. The traditional cognitive-developmental interpretation of Kohlberg and other scholars was based on an identification of this experience as *action and not judgment*. From the present perspective, this experience was action-judgment1 at time-context1 (action and judgment at first time point and first context). Likewise, during the interview after the encounter, the report of participants in favor of helping was identified as *judgment and not action*. From the present perspective, this experience was action-judgment2 at time-context2 (action and judgment at second time point and second context). At each point in time, at each context, individuals’ actions reflected their judgment; they acted as they judged. For many participants, how they judged (and acted) in the first context was different from how they judged (and acted) in the second context. The tasks were different; it is likely that for many people, the strengths of and the coordination between multiple moral motivations in each context were different. There was contextual and temporal variation in rather than a discrepancy between judgment and action. This is an application of iterative view of the human mind that takes into account time, context and variability (Lichtwarck-Aschoff et al., 2008; Thelen and Smith, 2006; van Geert, 2009).

In the foregoing example, moral judgments were different as a result of different syntheses of or compromise between multiple moral motivations in different contexts. The emergence, salience and strength of the motivation of self-interest (Greene, 2013; Haidt, 2012) is likely to be stronger when participants are directly asked for help by a person in need. Piaget (1932) differentiated such experiences as representing “effective moral thought, ‘moral experience’” for the child: “It is that which leads him to form such moral judgments as will guide him in each particular case as it comes his way and enable him to evaluate other people’s actions when these concern him more or less directly” (p. 171). This *practical morality* is contrasted with “theoretical or verbal moral thought,” which “appears whenever the child is called upon to judge other people’s action that do not interest him directly” (p. 171; see also Kohlberg, 1971).

## The construction of self-interest and developmental transformations

Throughout life-span development, the personal power of the individual increases by virtue of her participation in social institutions and activities are supportive (Dewey and Tufts, 1910, p. 11). In turn, the mind becomes more social by a transformation of self-interest on behalf of the person’s identification with the psychological surrounding (Kegan, 1994): society becomes part of the individual as the individual becomes part of society. In this process of socialization, self-interest “inevitably undergoes a transformation” (Dewey and Tufts, 1910, p. 11). Other people’s interests become more coordinated and balanced with “those interests that center in my more individual good. Conscious egoism and altruism become possible” (p. 11).

A simple form of self-interest has been directly represented in Kohlberg’s theory by the preconventional level, and more specifically by stage 2. A more complex form of self-interest is found at the conventional level based on an identification with interpersonal relationships (stage 3), and social order (stage 4). Postconventional morality represents a more complex and psychologically *adequate* (Kohlberg and Mayer, 1972) construction of self-interest based on the greatest good for the greatest number (stage 5) or self-chosen universal principles of justice in one’s conscience (stage 6). Dewey and Tufts (1910) characterized this new construction of self-interest with high autonomy based on voluntary choice: “If the people really works out a higher type of conscious and personal morality, it means not only a more powerful individual, but a reconstructed individual and a reconstructed society” (p. 89).

Kohlberg (1971) asserted that “moral judgment entails a concern for welfare consequences” (p. 191). This emphasis stressed the individual’s concern for the welfare of others. While this is important, the present motivation represents the person’s concern for his own welfare, as a fundamental motivation that affects moral decision making. While the cognitive-developmental perspective reserved this motivation almost exclusively for preconventional morality, the present dynamic systems perspective views it to be ubiquitous in everyday life, even in the experiences of people traditionally considered to be at higher levels of development. In the dynamic system of moral motivation, self-interest is not isolated from but complementary to more altruistic concerns. This dynamic, dialectic and relational view is consistent with the moral philosophy of Nietzsche (1986, 2002) in the sense that what people perceive and construct as good and evil are not exclusive entities ontologically, but rather inextricably interconnected with each other.

Self-interest is a broad category of personal inclinations, needs, wishes, desires, preferences, and beliefs that are psychologically constructed and experienced to be essential to the survival and welfare of the individual. Self-interest includes physical needs of the organism such as physiological and safety needs (Maslow, 1954), psychological needs such as relatedness, competence and autonomy (Deci and Ryan, 2000), as well as the psychological experiences (such as beliefs) that the self identifies with Kegan (1994) and Kegan and Lahey (2009). Self-interest also involves goals that the individuals have for the protection and enhancement of personal life conditions. This broad category acquires specificity in the context of a moral dilemma, which evokes only its relevant aspects. Each moral dilemma places a demand on self-interest. The aspects of self-interest that are involved in the moral judgment and action process depend on how individuals construct the dilemma to be relevant to their personal experience. In turn, “self-interest makes the judger alert to certain aspects of the situation judged” (Dewey, 1903, p. 132). This selective attention is a key mechanism by which self-interest affects moral judgment.

As a motivational tendency, self-interest reflects the constructive nature of moral judgment and action. That is, the motivational force at work in the formation of moral judgment is not an objective, standard or absolute measure of self-interest, but a subjective construction of perceived self-interest by the individual at a given point in time and context. In particular, the individual’s perception of the self-interest cost of moral action is a direct contributor to the emergence of this action. Each action possibility has a perceived cost to one’s own personal welfare. To the extent that the individual is willing and able to endure this perceived cost, the corresponding action is more likely to emerge. If the individual is unwilling or unable to bear the perceived self-sacrifice that is associated with an anticipated action, this action is not likely to emerge.

It is important to note that this capacity or willingness to endure self-sacrifice is influenced by the operation of other motivations, namely, justice and care. For example, the intensification of the feeling of compassion toward someone in need amplifies this capacity, and thus, increases the likelihood of emergence of altruistic actions that otherwise would have been inhibited by concerns of self-sacrifice.

In the formation of moral judgment, this motivation frequently represents the sacrifice that possible judgments and actions are likely to bring. Prior to the emergence of moral judgment and action, there are perceptions and mental evaluations of possible sacrifice associated with various options. These perceptions and evaluations are integral to moral decision making, determining which judgment and action will emerge. A key consideration in this process is whether and how the individual is willing and able to bear the sacrifice that a particular judgment and action may necessitate. Thus, moral judgment and action occur in the background of self-interest. For example, in many circumstances, honest action (e.g., admitting wrongdoing) requires relinquishing self-interest in various forms such as pride, status or personal advantage. Likewise, many prosocial actions such as altruism demand some degree of sacrifice from self-interest. Similarly, sociopolitical moral judgments (about individuals, their actions, or political events) are strongly influenced by our existing assumptions, inclinations, affiliations, and beliefs with which we have identified in our experience and construction of the self (e.g., defining oneself as a liberal, conservative, libertarian). Real-life moral judgment choices bring various costs to self-interest. The perception and evaluation of these possible costs are integral to the process of moral judgment. Moral dilemmas involve decisions about whether to avoid or endure certain costs, or the amount and the type of cost that the individual is ready and willing to experience for the sake of doing the right thing (e.g., helping someone in need by relinquishing existing personal advantage). To illuminate the complexity of real-life moral motivation, more attention should be paid in research to how people perceive and make sense of the personal costs of moral judgment and action.

## Sense of duty as moral motivation

Kant (1996) defined duty as “an action that is objectively practical according to this law, with the exclusion of every determining ground of inclination” (p. 205). On the other hand, he frequently used the terms “duty” and “the moral law” interchangeably: an action done out of “respect for the moral law” is an action done “from duty” (p. 205). Consistently, Kant presented moral law as a law of duty. Reason commands individuals to make “the thought of duty… the supreme *life-principle* of all morality in human beings” (p. 209).

Schopenhauer (1903) criticized Kant’s “imperative form of Ethics, i.e., the conception of obligation, of law, and of duty” as emerging from theology (p. 48). However, even though religious experience is a widespread source for the sense of duty; the proposition of duty as a central concept of morality does not have to rely on a religious foundation. Its justification can be entirely based on its essential value and function in human adaptation. Duty is ubiquitous. People frequently complete tasks with a sense of duty, however variable this sense may be in terms of its intensity and duration. The variability in people’s degree of dedication to tasks of everyday life is not evidence against the existence of a sense of duty. Both life-span development and everyday strivings are highly influenced by people’s sense of duty in terms of virtues or core strengths that are specific to developmental periods (Erikson and Erikson, 1997) or developmental tasks (Havighurst, 1956).

Kant (1996) presented the *observance* of duty as *virtue*. This is the process of dedication to duty and acting from a sense of duty as a motive (rather than from personal inclinations such as a desire to gain advantage). This process involves significant *sacrifices*. The organizing force of the sense of duty involves the experience of yielding or relinquishing personal desires, which otherwise contradict duty (Kant, 1996).

The experience can approximate the purity of moral motivation (acting from duty) to the extent that the individual *represents* “duty as connected with the sacrifices its observance (virtue) costs us rather than with the advantages it yields us” (Kant 1996, p. 282). Consistently, Kohlberg (1971) contended that “much morality involves basic sacrifice,” and that “a mature belief in moral principles in itself engenders a sacrifice of the rational ego, apart from other personality and emotional considerations” (p. 232).

Kant’s perspective resonates with Socrates’ conception of wisdom, which he identified with temperance. According to this conception, wisdom or temperance is not to be characterized by gaining any advantage, as it is “only the knowledge of knowledge and ignorance, and of nothing else” (Plato, 1892, Vol. 1, p. 36).

Despite the prevalence of self-interest, the foregoing philosophical perspectives represent a vision of human nature beyond its primacy. In line with this perspective, Lerner and Clayton (2011) questioned the *primacy of self-interest* by emphasizing strong emotions such as guilt and regret in the aftermath of self-interested behaviors. The present study examines justice and care in terms of duty.

## Justice as duty, and its relation to self-interest

A commitment to justice represents a particular motivational experience: justice as duty. This involves willingness to relinquishing some degree of self-interest and endure costs. As Glaucon asserted in Plato’s (1908) Republic “injustice is far more profitable to the individual than justice” (Plato, 1908, p. 54).

As a motive, justice represents a desire to recognize facts, fulfill promises and obligations (Mill, 2009). In terms of justice, Aristotle (1999) highlighted the importance of keeping one’s agreements (p. 68). According to Hobbes (1651) justice is a law of nature, based on fulfillment of agreements. Botes (2000) contended that “the ethics of justice is characterized by fairness and equality, rational decision-making based on universal rules and principles, and autonomous, impartial and objective decision-making” (Botes, 2000, p. 1074).

The postulation of justice as a distinct and universal motivation is a challenge to widespread paradigms in social sciences. For example, Kahneman et al. (1986) challenged the dominant view of human nature in economics based on self-interest. They found strong evidence for a tendency to protect fairness and react to unfairness, even at some cost to self-interest. Thus, “the preference that people have for being treated fairly and for treating others fairly” is a widespread phenomenon (Kahneman et al., 1996, p. S285). In addition, exploitative notwithstanding, “there is a significant incidence of cases in which firms, like individuals, are motivated by concerns of fairness” (Kahneman et al., 1986, p. S287).

In the dynamic system of moral motivation, multiple motivational tendencies form relationships as they change over time. In this process, they serve as contexts for each other’s development. For example, justice develops through encounters with self-interest. In this relational developmental process, boundaries between these motivational categories are not rigid or fixed, but rather flexible and fuzzy (van Geert, 2002, p. 321). Thus, as opposites, justice and self-interest are not mutually exclusive; rather, they are interconnected (Overton, 2015) in the totality of moral motivation as a developmental process. In this process, they penetrate and turn into one another (Hegel, 1952). Through repeated, iterative encounters with one another both can be transformed. Self-interest serves as a context for the experience and development of justice and vice versa. For example, violations of or threat to self-interest is a major stimulus for the experience and development of justice. Throughout development, the individual may construct and act on more inclusive conceptions of justice beyond the protection of immediate self-interest; and self-interest may become more comprehensive through the person’s identification with and concern for other people’s welfare.

Kals et al. (2001) conducted an empirical study on self-interest and justice for commitments in favor of and against environmental protection. They found that “a motive mixture of self-interest and justice motives” (p. 282) may be underlying commitments that endanger air quality. For such decisions, “self-interests are quite often competing with the ecological interests of society” (p. 282). According to the present perspective, self-interest may both compete and cooperate with justice (as duty). The same process is valid for care as duty, in its connection with self-interest, as explored in the next section. The relative strengths of competing and cooperating motives are variable interpersonally and intrapersonally, over time and across contexts.

As a motivational tendency, justice displays substantial intrapersonal and interpersonal variability. An individual values different facts over time and across contexts. Furthermore, different people value facts in different degrees; and they wish different facts to be recognized and accepted. While justice may operate as duty, its strength is highly variable. A major source of variation is the relation of self-interest to justice. The construction of self-interest may influence the degree to which facts are valued, and the kinds of facts that are valued. Nevertheless, in a developmental process, justice can acquire autonomy and serve as a governing principle for human action, in combination with and in relation to other motives. The notion of justice as duty reflects this possibility. The same notion holds for care as a distinct motive.

## Care as duty, and its relation to self-interest

The centrality of care in ethics has been proposed in the context of the relational nature of human experience in terms of interpersonal connections (Held, 2015; Tronto, 2015). Thus, care has been proposed as an essential human need (Gilligan, 1977, 1982; Marshall, 2019).

Care corresponds to one of the *passions* that Hobbes (1651) proposed: “a fellow-feeling” arising “from the imagination that” someone else’s “calamity may befall” oneself (p. 37). Hobbes identified this experience as *compassion*. According to Hobbes, this was one of the many passions. On the other hand, Schopenhauer (1903) asserted compassion as the essence of morality.

Corollary to Lerner and Clayton’s (2011) *justice imperative*, there is a *care imperative*, the violation of which can have significant psychological and social costs. This is care as duty. The care imperative is reflected in Schopenhauer’s (1903) core principle of *compassion*, as the *foundation of morality*: “Do harm to no one; but rather help all people, as far as lies in your power” (p. 54).

As Marshall (2019) pointed out, care is not only “a source of meaning and fulfillment in life,” but also “often messy, ambiguous, frustrating, challenging, exhausting and personally costly” (p. 182). From the present dynamic systems framework, the complexity of and challenges to care have to do with its relationships with other moral motivations such as self-interest and justice. That is, moral adaptation to life is a developmental process of adequately regulating multiple motives. Thus, a key developmental task is to be able to effectively regulate the relationships of care to other motivational tendencies.

Care may have some basis in group life, particularly in terms of the necessity of cooperation for survival and safety. Care may have an origin in what Dewey and Tufts (1910) called “the gregarious instinct,” which “may be the most elemental of the impulses which bind the group together” (p. 35). Consequently, care may be “reinforced by sympathies and sentiments growing out of common life, common work, common danger, common religion” (p. 35). This proposition resonates with Greene’s (2013) contention that the human brain has evolved to curb selfishness and establish in-group solidarity and cooperation, motivated in part by the needs to protect against and compete with outgroup forces.

The distinction between self-interest and care is reflected in Mill’s (2009) characterization of selfishness, which represents “caring for nobody but themselves” in contrast to “a fellow-feeling with the collective interests of mankind” (p. 26). Such a distinction was reflected in the assessment of Cropanzano et al. (2005) in their attempt to identify motives other than self-interest: “If an act is intended to benefit another person in any part, it is not exclusively self-interested” (p. 985). This implies that an act can be motivated partly by self-interest and partly by care. Depending on the relationship between these two motivational tendencies, including their relative strengths, the motivational experience of the individual and the manifested action and judgment can be variable.

Cropanzano et al. (2005) explored self-interest as “an important human motive,” but also challenged its presumed monopoly in human behavior by distinguishing and identifying other motives such as “empathy toward others and adherence to moral duty” (p. 985). They cited evidence from economics that people’s behavior is not always explained by “monetary payouts”; and researchers appealed to other possible factors such as “cooperation,” “fairness,” and “enforcement of norms” (p. 986). Similarly, individuals can act to help others in the absence of apparent immediate personal profit, and even at great costs to their benefits (pp. 986–987). However, altruism can be viewed as consistent with a broader conception of self-interest based on its well-being benefits for helpers. On the other hand, citing Batson’s (1995) empathic-altruism hypothesis, Cropanzano et al. (2005) contended that “empathic individuals experience and care about the predicament of others” and thus, “the overarching goal” of empathic altruism “is not self-interested” (p. 986).

According to Batson’s (1995) research and the assessment of Cropanzano et al., when empathy is prevalent, helping behavior is more likely to be motivated by considerations of the other person’s needs and interest. According to the present model, this represents the strength of care as a motive. By contrast, when “generalized distress” was prevalent, people “tended to take the most efficient route to mood enhancement,” by helping “if necessary,” but choosing “to escape when given an opportunity” (Cropanzano et al., 2005, p. 987). In the latter case, self-interest prevails in its relationship with care. Aristotle’s (1935); Aristotle’s (1999) presentation of friendship for utility and friendship for pleasure characterizes two common forms of operation of the self-interest as motivation in relation to care. In both forms, self-interest overpowers and rules over the motivation of care. Friendship serves as a means for the primary aim of fulfilling self-interest.

Caring for others may support caring for oneself. However, when this relationship is taken to an extreme degree on behalf of self-interest, then care may be substantially compromised. That is, when self-interest dominates this relationship, the duty of care may be sacrificed for the sake of self-interest. If the individual is not able to unite or reconcile the satisfaction of self-interest and the fulfillment of key ethical duties such as caring for loved ones, then he may resort to the pursuit of self-interest at the expense of the duty of care.

Alternatively, self-interest may be employed in the service of care. This relationship may utilize the neurobiological basis of care in empathy; witnessing other people’s suffering evokes distress in people (Greene, 2013). In the experience of caring for other people in pain, the genuine desire to help is interconnected with individuals’ self-interested motives to reduce their own distress. One’s own distress may be used as a catalyst to extend and strengthen care. Another form of this relationship may involve self-sacrifice, as self-interest may be relinquished for the sake of care. Such a sacrifice of self-interest for the sake of care is an aspect of how “an individual chooses rationally only in so far as he constrains his pursuit of his own interest or advantage to conform to principles expressing the impartiality characteristic of morality” (Gauthier, 1986, p. 4).

## Conclusion

Real-life experience of moral judgment and action is motivated, and moral motivation involves multiple forces, subject to self-organization and self-regulation. There are competitive and cooperative relationships between multiple motivations. Out of these relationships moral judgment and action arise. Moral motivation operates and changes in an iterative process over time, building on previous states, and in close connection with contextual factors. As broad categories of moral motivation, self-interest and duty serve as fundamental motivational forces, which are connected with each other in competitive and supportive relationships. The development of moral motivation involves regulation of self-interest and duty, including constraining or relinquishing certain forms and experiences of self-interest that may be impeding rather than facilitating adaptation to life.

In this paper, a dialectic and relational view of moral motivation was presented by incorporating insights from moral philosophy, moral psychology and the process-relational metatheory. Two major forms of duty were examined in relation to self-interest: justice as duty, and care as duty. This view has theoretical and practical implications toward both understanding and addressing complex moral phenomena, including moral conflicts and moral development. Theoretical implications involve shifts toward more adequate conceptions and accounts in moral psychology that represent real-life complexity of moral judgment, action, motivation and development. In keeping with Lewin’s (1943) suggestion “there is nothing as practice as a good theory” (p. 118), such theoretical advances toward real-life moral complexity are likely to have practical benefits toward addressing moral conflict and promoting increasingly adaptive moral motivations throughout the lifespan. Such advances can shed light on the psychological and environmental conditions under which the sense of duty can be strengthened and self-interest can be effectively regulated in the dynamic complexity of moral motivation. Building on the present CDS perspective, future studies can explicate and empirically explore supportive and competitive relationships between justice, care and self-interest.

## Data Availability

The original contributions presented in the study are included in the article/supplementary material, further inquiries can be directed to the corresponding author.
